# An investigation on fermentative profile, microbial numbers, bacterial community diversity and their predicted metabolic characteristics of Sudangrass (*Sorghum sudanense* Stapf.) silages

**DOI:** 10.5713/ab.21.0326

**Published:** 2022-01-04

**Authors:** Siran Wang, Junfeng Li, Jie Zhao, Zhihao Dong, Tao Shao

**Affiliations:** 1 Institute of Ensiling and Processing of Grass, College of Agro-Grassland Science, Nanjing Agricultural University, Nanjing 210095, China

**Keywords:** Bacterial Community, Fermentation Quality, Metabolic Characteristics, Silage, Sudangrass

## Abstract

**Objective:**

This study aimed to investigate the fermentation profiles, bacterial community and predicted metabolic characteristics of Sudangrass (*Sorghum sudanense* Stapf.) during ensiling.

**Methods:**

First-cutting Sudangrass was harvested at the vegetative stage and ensiled in laboratory-scale silos (1 L capacity). Triplicate silos were sampled after 1, 3, 7, 15, 30, and 60 days of ensiling, respectively. The bacterial communities on day 3 and 60 were assessed through high-throughput sequencing technology, and 16S rRNA-gene predicted functional profiles were analyzed according to the Kyoto encyclopedia of genes and genomes using Tax4Fun.

**Results:**

The Sudangrass silages showed good fermentation quality, indicated by higher lactic acid contents, and lower pH, butyric acid and ammonia nitrogen contents. The dominant genus *Lactococcus* on day 3 was replaced by *Lactobacillus* on day 60. The metabolism of amino acid, energy, cofactors and vitamins was restricted, and metabolism of nucleotide and carbohydrate was promoted after ensiling. The 1-phosphofructokinase and pyruvate kinase of bacterial community seemed to play important roles in stimulating the lactic acid fermentation, and the promotion of arginine deiminase could help lactic acid bacteria to tolerate the acidic environment.

**Conclusion:**

High-throughput sequencing technology combined with 16S rRNA gene-predicted functional analyses revealed the differences during the early and late stages of Sudangrass ensiling not only for distinct bacterial community but also for specific functional metabolites. The results could provide a comprehensive insight into bacterial community and metabolic characteristics to further improve the silage quality.

## INTRODUCTION

Silage making has become a major method of forage conservation worldwide. The epiphytic lactic acid bacteria (LAB) present on forage crops and grasses convert water-soluble carbohydrates (WSC) into lactic acid during the fermentation, thereby reducing the final pH and enhancing the nutritive value of the silage [1]. Benefiting from this acidification process, silages can be stored for a long time with reduced risk of decay. Nevertheless, unsuitable ensiling conditions might facilitate clostridial activity and produce large amounts of ammonia nitrogen and butyric acid, thus resulting in poor quality of silage.

Sudangrass (*Sorghum sudanense* (Piper) Stapf.) is a fine annual forage of Gramineae, Sorghum Genus that thrives in warm natural environment, with strong resistance against drought and barren soil, high yield, and strong regeneration capability [2]. It can be used for livestock, aquaculture foods, and protecting fishing ponds, especially in arid and semiarid regions [3]. However, as a promising tropical forage, the exploration of bacterial compositions in Sudangrass silages fall behind that of other forage crops, such as Italian ryegrass [4,5], alfalfa [6,7] and maize [8,9]. Characterizing the bacterial community during the ensiling could provide insights into approaches to further improve the fermentation quality of Sudangrass silages.

Recently, the high-throughput sequencing technology combined with 16S rRNA gene-predicted functional analyses has been widely used in many researches to describe the changes of bacterial community and their metabolic pathways during ensiling. Gharechahi et al [8] evaluated the dynamic behavior of bacterial community in maize silages, and found that the functional metagenome prediction exhibited a connection between the ensiling process and enrichment of pathways for propionate and bile acid metabolism and those for degradation of toxic compounds. Their results proved that the microbes in silages played a crucial role in detoxifying the plant-derived toxic metabolites. Wang et al [10] investigated the 16S rRNA gene-predicted functional profiles of *Moringa oleifera* leaf silage, and reported that the metabolism of amino acid including proline, serine, alanine, threonine, glycine and arginine was closely correlated with the formation of ammonia nitrogen in silages. Du et al [11] assessed the bacterial community structure and metabolic gene clusters during the ensiling of paper mulberry, and concluded that amino acid metabolism and carbohydrate metabolism both played critical roles in affecting the final fermentation product of silage. However, to the best of our knowledge, there is limited information about the bacterial community and their 16S rRNA gene-predicted functional profiles in Sudangrass silages. Also, most researches just investigated the changes of fermentative parameters of silage without analyzing the complicated bacterial community dynamics, bacterial interactions and their functional shifts.

Therefore, the purpose of this study was to evaluate the fermentative profiles, microbial populations, bacterial community dynamics and 16S rRNA gene-predicted functional characteristics during the ensiling of Sudangrass.

## MATERIALS AND METHODS

### Silage preparation

Eighteen 120 m^2^ plots of Sudangrass (*Sorghum sudanense* (Piper) Stapf.) grew in the experimental field of Nanjing Agricultural University. This area has a subtropical monsoon climate with an average temperature of 15.7°C, average elevation of 24.8 m and mean annual precipitation of 1,105 mm. The soil type in this region belongs to clay loam. Six plots of Sudangrass were randomly chosen and collected at the vegetative stage (height: 1.7 to 2.0 m) for silage making. The chemical parameters and microbial counts of fresh Sudangrass are described in Table 1. Sudangrass was cut by a forage chopper (93ZT-300; Xingrong Co. Ltd, Guangzhou, China) to a theoretical 2 to 3 cm length. Without any additives or wilting, approximately 700 g of chopped fresh Sudangrass was packed in a plastic silo (1 L capacity). Silages were conserved at ambient temperature (20°C to 30°C), and ensiling was performed in triplicate. The silos were opened after 1, 3, 7, 15, 30, and 60 days of ensiling for analyzing the fermentation parameters and bacterial community.

### Chemical analysis

When taking out samples, the whole content of each silo was mixed uniformly in a clean plastic container. Thirty-five gram of fresh forage or silage was mixed with 70 mL of distilled water and preserved at 4°C for 24 hours. Then we filtered the extracts using a filter paper and two layers of cheesecloth. The buffering capacity of fresh forage was determined according to the method of Playne and McDonald [12]. The pH of fresh forage or silage was determined by a glass electrode pH meter. Then, the filtrate was stored at −20°C for analyzing ethanol, organic acids and ammonia nitrogen (NH_3_-N). The organic acid and ethanol contents were analyzed using the Agilent HPLC 1260 (Agilent Technologies, Inc., Santa Clara, CA, USA; column: Carbomix H-NP5, Sepax Technologies, Inc., Newark, DE, USA; detector: refractive index detector, Agilent Technologies, Inc., USA; eluent: 2.5 mmol/L H_2_SO_4_, 0.5 mL/min; temperature: 55°C). The NH_3_-N contents were determined based on the description of Broderick and Kang [13].

One-hundred grams of fresh forage or silage was freeze-dried to determine dry matter (DM), and then milled to pass a 1 mm sieve for later analysis. The milled sample was used for the following analyses. The contents of total nitrogen were measured according to Kjeldahl method [14]. The WSC contents were determined using anthrone colorimetry method [15]. The acid detergent fiber, acid detergent lignin, and neutral detergent fiber contents of fresh material were determined by the method of Van Soest et al [16].

For enumeration of the microorganisms, 10 g pre-ensiled sample or silage was shaken well with 90 mL of sterilized saline solution at 120 rpm for 2 h. Then 1 mL solution was used for 10-fold serial dilution for microorganism counting, and then the remaining solution was filtered through 4 layers of medical gauze and stored in the −80°C refrigerator for DNA extraction. The colonies of LAB were counted on MRS agar (de Man Rogosa Sharpe agar) medium after incubation in an anaerobic incubator (N_2_:H_2_:CO_2_ = 85:5:10, YQX-II; CIMO Medical Instrument Manufacturing Co., Ltd., Shanghai, China) at 30°C for 3 days. Aerobic bacteria were cultured and counted on nutrient agar medium (Nissuiseiyaku Ltd., Tokyo, Japan). Yeasts were counted on potato dextrose agar (Nissuiseiyaku Ltd., Japan) and acidified with sterilized tartaric acid solution to pH 3.5. These agar plates were incubated at 30°C for 3 days. Enterobacteriaceae was counted on the Violet Red Bile Glucose Agar medium after 24 h of incubation at 37°C under aerobic conditions. The microbial data were obtained as colony-forming units (cfu) and were transformed to a logarithmic scale on a fresh weight (FW) basis.

### High-throughput sequencing analysis

Fresh Sudangrass (SDFM) and silage samples on day 3 (SD-3) and day 60 (SD-60) were chosen for analyzing the bacterial community using high-throughput sequencing. The solution for DNA extraction was centrifuged at 10,000×g for 15 min to obtain a pellet for subsequent DNA extraction, which was conducted using the FastDNA SPIN Kit and the FastPrep Instrument (MP Biomedicals, Santa Ana, CA, USA) according to the manufacture’s protocols. The quantity and quality of DNA were evaluated by NanoDrop 2000 UV-vis spectrophotometer (Thermo Scientific, Wilmington, DE, USA).

The V3–V4 region of the bacterial 16S ribosomal RNA gene was amplified by polymerase chain reaction (PCR) using the primers 338F and 806R [17]. The PCR products were purified using the AxyPrep DNA Gel Extraction Kit (Axygen Biosciences, Union City, CA, USA) and quantified using QuantiFluor-ST (Promega, Madison, WI, USA) according to the manufacturer’s protocol. The DNA samples were paired-end sequenced on an Illumina MiSeq PE300 platform (Illumina Inc., San Diego, CA, USA) at Majorbio Bio-Pharm Technology Co., Ltd. (Shanghai, China).

All the raw reads were checked using FLASH (version 1.2.11), and low-quality sequences (quality scores below 20) were discarded according to the QIIME quality control process (version 1.7.0). Operational taxonomic units (OTUs) were first clustered with a 97% similarity cutoff using UPARSE pipeline (version 7.0 http://drive5.com/uparse/). Then the chimeric sequences were identified and removed using UCHIME (uparse-cluster otus meta derepprefixsorted. fasta-otusotu_0.97/cluster.fasta-otu_radius_pct3). The alpha-diversities including OTUs, Shannon, Chao1, Sobs, Simpson, Ace, and Coverage indexes were calculated using Mothur (version 1.30.1). The Venn diagram was obtained using Venn Diagram software. Community structure was characterized at the phylum and genus levels using the Silva database (Release132 http://www.arb-silva.de) with a confidence threshold of 70%. The metabolic potential of the bacterial community and the composition of functional genes were postulated by assigning 16S rRNA marker gene sequences to functional annotations of sequenced metagenomic sequences based on the Kyoto encyclopedia of genes and genomes (KEGG) on the first, second and third pathway levels, using Tax4Fun (version 0.3.1) as described by Kathrin et al [18].

The sequencing data reported in this study has been deposited in the National Center for Biotechnology Information sequence read archive database under the accession number PRJNA739859.

### Statistical analysis

The Statistical Packages for the Social Sciences (SPSS, version17.0) were used for data analysis. Data on microbial populations, fermentation parameters, chemical compositions, and relative abundances of bacterial community compositions, predicted metabolic pathways and key enzymes in Sudangrass silages was subjected to one-way analysis of variance (ANOVA). Statistical differences among means were measured through Tukey’s multiple comparison. Differences were regarded significant at p<0.05.

## RESULTS

Changes in fermentation characteristics and microbial compositions during the ensiling of Sudangrass are described in Table 2. The pH decreased (p<0.001) from 6.09 in fresh Sudangrass to 4.85 on day (d) 3, and a large reduction (p<0.001) to 4.25 on d 15, and then pH remained at this level until d 60 of ensiling. The lactic acid contents significantly (p<0.001) increased from 5.78 to 25.4 g/kg DM during the initial 7 days of fermentation, and reached the maximum value (62.6 g/kg DM) on day 60. The ratio of lactic acid to acetic acid tended to increase during the whole ensiling process. The relatively stable levels in propionic acid, isobutyric acid and butyric acid concentrations were observed during ensiling. Ethanol contents increased rapidly from 17.5 g/kg DM on d 1 to 30.9 g/kg DM on day 3, and reached the maximum content (42.5 g/kg DM) on day 30, and decreased to 40.0 g/kg DM on day 60. The NH_3_-N concentrations continuously increased to the maximum value (56.7 g/kg total nitrogen [TN]) on d 60. The LAB populations significantly (p<0.001) increased from 7.45 log_10_ cfu/g FW on d 1 to the highest value (9.71 log_10_ cfu/g FW) on d 7, and then gradually decreased to 6.22 log_10_ cfu/g FW on d 60. The Enterobacteriaceae populations decreased largely within initial 15 days to 5.36 log_10_ cfu/g FW as compared with the fresh Sudangrass (7.27 log_10_ cfu/g FW), and then exhibited a gradual decline until the final stage of fermentation. The yeast populations significantly (p<0.001) decreased from 4.72 log_10_ cfu/g FW on d 1 to the lowest value (3.91 log_10_ cfu/g FW) on d 15, and then gradually increased to 4.49 log_10_ cfu/g FW on d 60.

**Figure 1:**
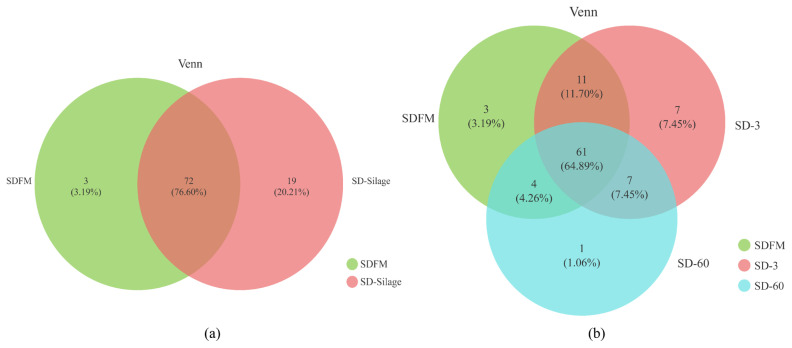
Venn diagram of the core OTUs between the fresh and silages (a) and Venn diagram of the core OTUs among fresh material, day 3 and 60 silages (b). SDFM, fresh Sudangrass; SD-Silage, Sudangrass silage after 3 and 60 days; SD-3, Sudangrass silage after 3 days of ensiling; SD-60, Sudangrass silage after 60 days of ensiling.

The diversity and richness indexes of bacterial communities in fresh Sudangrass and Sudangrass silages on d 3 and 60 are illustrated in Table 3. The indexes of Shannon and sobs in SDFM were higher than that of SD-3 and SD-60. SD-3 had relatively higher indexes of OTUs, Shannon, Chao1, sobs and Ace, but lower indexes of Simpson compared with SD-60. All the samples had higher coverage indexes (>99.97%). The Venn diagram was exhibited in Figure 1. The overlapping OTUs (72) between SDFM and SD-Silage had 76.60% of total OTUs. The overlapping OTUs (11) between SDFM and SD-3 were relatively higher than the overlapping OTUs (7) between SD-3 and SD-60.

**Figure 2:**
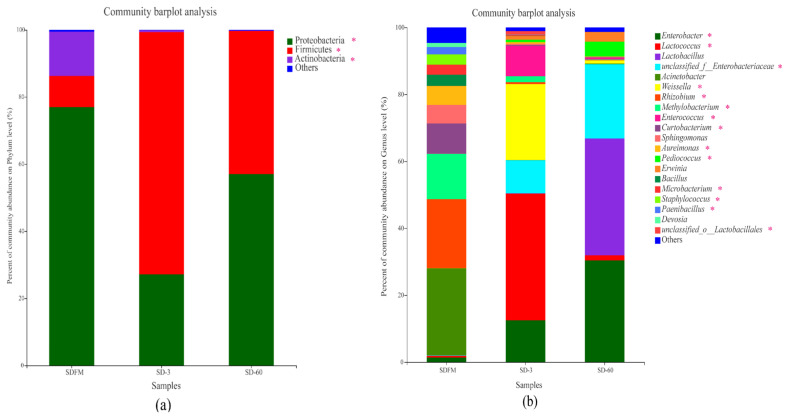
Compositions of the bacterial community at phylum (a) and genus (b) levels in fresh Sudangrass and Sudangrass silages. SDFM, fresh Sudangrass; SD-3, Sudangrass silage after 3 days of ensiling; SD-60, Sudangrass silage after 60 days of ensiling. * 0.01<p≤0.05.

Phylum level compositions of the bacterial community in fresh Sudangrass and Sudangrass silages are shown in Figure 2a. Proteobacteria was the most predominant phylum (77.0%) in fresh Sudangrass, followed by Actinobacteria (13.2%) and Firmicutes (9.31%). The most abundant phylum during the initial 3 days of fermentation was Firmicutes (72.2%), while decreased to 42.6% at the end of ensiling. After ensiling, the Proteobacteria accounted for 27.2% and 57.0% on d 3 and 60, respectively.

Genus level compositions of the bacterial community in fresh Sudangrass and Sudangrass silages are described in Figure 2b. The main dominant genera in SDFM were *Acinetobacter* (26.01%) and *Methylobacterium* (13.58%). At the initial stage of ensiling, *Lactococcus* (37.92%) rapidly became predominant on d 3, followed by *Weissella* (22.69%), *Enterobacter* (12.48%) and Enterobacteriaceae (9.89%). At the end of ensiling, the dominant role of *Lactococcus* was replaced by *Lactobacillus* (34.91%) on d 60, followed by *Enterobacter* (30.38%) and Enterobacteriaceae (22.17%).

**Figure 3:**
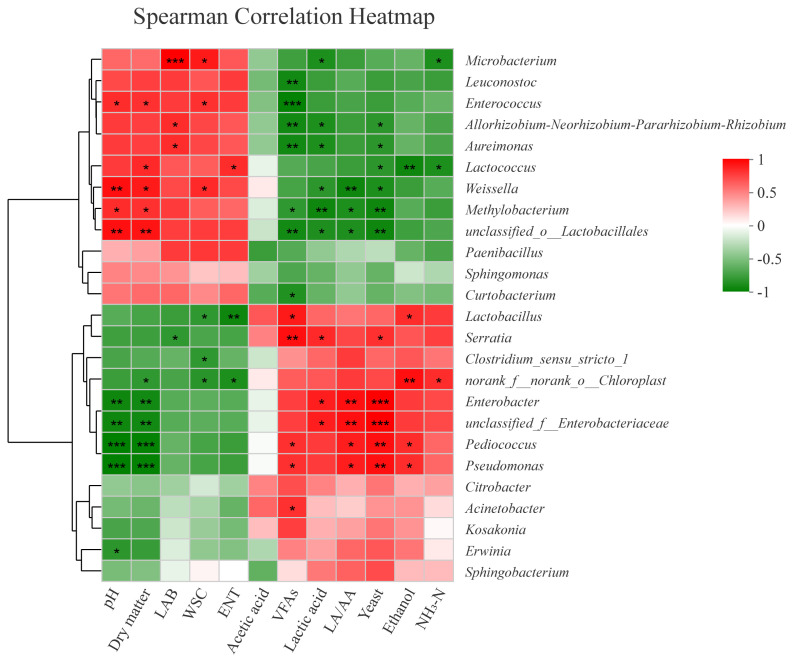
Spearman correlation heatmap of bacterial genera and fermentation parameters of Sudangrass silages after 3 and 60 days of ensiling. The scale colors denote whether the correlation is positive (closer to 1, red squares) or negative (closer to −1, green squares) between the taxa and the production parameters. NH3-N, ammonia nitrogen; LA/AA, the ratio of lactic acid to acetic acid; VFAs, volatile fatty acids; WSC, water soluble carbohydrate; LAB, lactic acid bacteria; ENT, Enterobacteriaceae. * 0.01<p≤0.05; ** 0.001<p≤0.01; *** p≤0.001.

The correlations between fermentation parameters and bacterial abundance of Sudangrass silages are illustrated by spearman correlation heatmap in Figure 3. *Lactobacillus* was positively connected with contents of volatile fatty acids and ethanol, while negatively related to Enterobacteriaceae populations and water soluble carbohydrate contents.

**Figure 4:**
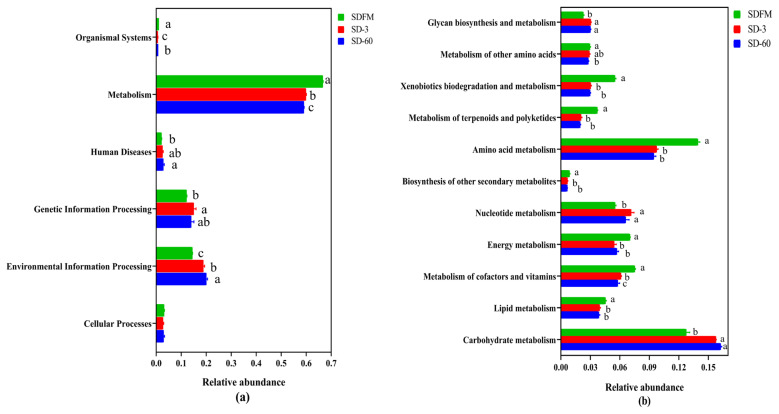
Bar graphs showing statistical differences of 16S rRNA gene-predicted functional profiles on the first (a) and second (b) pathway levels obtained with Tax4Fun. SDFM, fresh Sudangrass; SD-3, Sudangrass silage after 3 days of ensiling; SD-60, Sudangrass silage after 60 days of ensiling. ^a–c^ Means within the same parameter with different letters differ significantly from each other (p<0.05).

The statistical differences of 16S rRNA gene-predicted functional profiles on the first and second pathway levels are described in Figure 4. As shown in Figure 4a, the relative abundance of metabolism was obviously higher than other pathways, while the metabolism was inhibited after ensiling. As described in Figure 4b, the metabolism of amino acid and carbohydrate was much higher than other pathways. The amino acid metabolism, energy metabolism, and metabolism of cofactors and vitamins were significantly (p<0.05) reduced, while the nucleotide metabolism and carbohydrate metabolism were significantly (p<0.05) enhanced after ensiling.

**Figure 5:**
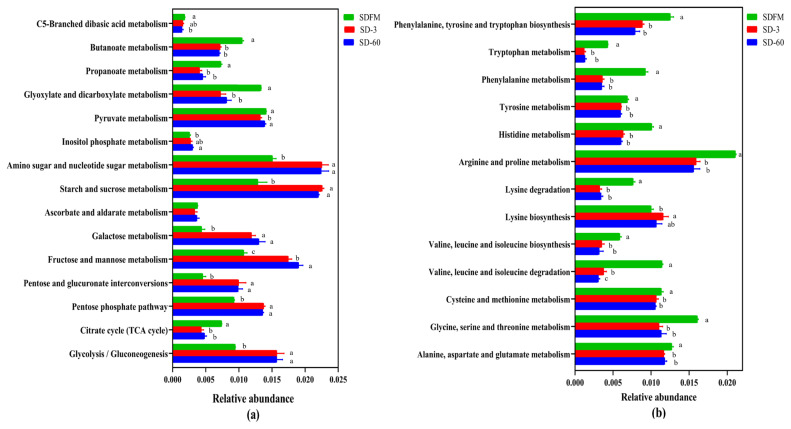
Bar graphs showing statistical differences of 16S rRNA gene-predicted carbohydrate metabolism (a) and amino acid metabolism (b) on the third pathway level obtained with Tax4Fun. SDFM, fresh Sudangrass; SD-3, Sudangrass silage after 3 days of ensiling; SD-60, Sudangrass silage after 60 days of ensiling. ^a,b^ Means within the same parameter with different letters differ significantly from each other (p<0.05).

The statistical differences of 16S rRNA gene-predicted carbohydrate and amino acid metabolism on the third pathway level are illustrated in Figure 5. Many of carbohydrate metabolic pathways were significantly (p<0.05) promoted after ensiling, while the metabolism of butanoate, propanoate, glyoxylate and dicarboxylate, and citrate cycle (tricarboxylic acid [TCA] cycle) was significantly (p<0.05) inhibited after ensiling. Many of amino acid metabolic pathways were significantly (p<0.05) reduced after ensiling.

**Figure 6:**
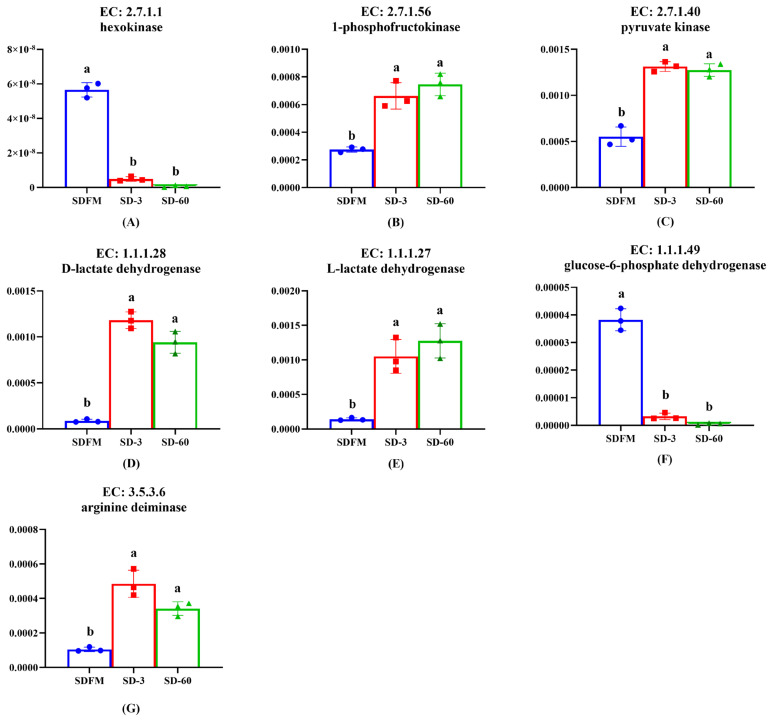
Changes of key enzymes involved in bacterial community metabolism during the ensiling of Sudangrass. EC, reference metabolic pathway highlighting numbers; SDFM, fresh Sudangrass; SD-3, Sudangrass silage after 3 days of ensiling; SD-60, Sudangrass silage after 60 days of ensiling. ^a,b^ Means within the same parameter with different letters differ significantly from each other (p<0.05).

As shown in Figure 6, the relative abundances of hexokinase and glucose-6-phosphate dehydrogenase were significantly (p<0.005) decreased after ensiling. In contrast, the relative abundances of 1-phosphofructokinase, pyruvate kinase, D-lactate dehydrogenase, L-lactate dehydrogenase, and arginine deiminase were significantly (p<0.035) enhanced after ensiling.

## DISCUSSION

### Fermentation characteristics and microbial compositions during ensiling

In this study, the pH values dropped and lactic acid contents increased rapidly during the first 7 days, which indicated extensive lactic acid-fermentation occurred at the initial stage. The ratio of lactic acid to acetic acid in silage that is greater than 3.0 is known as homolactic fermentation whereas less than 3.0 is heterolactic [19]. Herein, the ratios of lactic acid to acetic acid were always higher than 3.0, substantiating a stronger homo-fermentation during the ensiling of Sudangrass. Trace amounts of propionic acid, isobutyric acid and butyric acid were determined during the fermentation period. It was ascribed to a quick decline in pH values due to the rapid generation of lactic acid, suppressing the growth of clostridia and other undesirable microorganisms. Ethanol is considered as an undesirable product in preserving forage because it causes extremely higher DM and energy losses. According to the description of Kung et al [20], over 30 to 40 g/kg DM of ethanol content may be associated with the action of yeast. In this study, ethanol contents were higher than 30.0 g/kg DM on d 3, suggesting that ethanol was mainly produced by yeasts and other microbes (e.g. hetero-LAB). Typically, NH_3_-N contents of quality silages should be less than 100 g/kg TN [21]. Herein, the NH_3_-N contents satisfied that requirement (<57.0 g/kg TN), which may be associated with the rapid decrease in pH at early stage.

After 15 days of ensiling, the decreased populations of LAB might be due to the insufficient substrates and acidic environment in silages. The decreased populations of Enterobacteriaceae during fermentation was thus probably due to the rapid acidification at the early stage. The increased populations of yeast in silages after 15 days was possibly because many yeasts could grow under the acidic conditions, even at pH 3.5 [22].

### Bacterial community compositions in fresh material and silages

The higher indexes of OTUs, Shannon, Chao1, Sobs and Ace in SDFM and SD-3 than SD-60 indicated that a more abundant bacterial community existed in SDFM and SD-3 than SD-60. The lower Shannon and higher Simpson indexes in SD-3 indicated that the anaerobic environment could rapidly reduce the bacterial richness and diversity in Sudangrass silages at the early stage of fermentation. Over 99.97% of the coverage in all samples suggested that the sequencing process was adequate to describe the dynamic changes of the bacterial community. The higher proportions of distinct OTUs in SD-Silage group suggested that there was a remarkable difference in bacterial community between fresh Sudangrass and Sudangrass silages. The relatively higher ratios of overlapping OTUs between SDFM and SD-3 than that between SD-3 and SD-60 indicated that the bacterial community in Sudangrass silages varied dramatically during the entire ensiling process.

Once ensiling, the most dominant phylum of Proteobacteria on fresh Sudangrass was quickly substituted by Firmicutes. Similarly, Romero et al [23] found that the predominant phylum was converted from Proteobacteria (84%) on raw material to Firmicutes (86.0%) in silages. The microbes belonging to Firmicutes are crucial acid-hydrolytic microbes under anaerobic circumstances containing anaerobic rumens and reactors, whilst they could produce various proteases, lipases, cellulases and other enzymes. The acidic and anaerobic environments during ensiling benefit the growth of Firmicutes [24]. In this study, the rapid change of predominant phylum from Proteobacteria to Firmicutes within 3 days was mainly due to the formation of anaerobic environment in Sudangrass silages. It’s worth noting that a higher proportion of Actinobacteria was noticed in fresh Sudangrass. Actinobacteria were widely distributed in both terrestrial and aquatic ecosystems, especially in soil, and they exhibited diversely physiological and metabolic properties, such as the production of extracellular enzymes and the formation of various secondary metabolites [25]. Herein, the higher ratios of Actinobacteria in SDFM may be connected with the soil contamination when harvesting the Sudangrass.

The most abundant genus in SDFM was *Acinetobacter*. After ensiling, the anaerobic condition inhibited the growth of *Acinetobacter* since they are non-fermentative and strictly aerobic bacteria. Besides, SDFM had a higher abundance of *Methylobacterium*. *Methylobacterium* was reported to include species of facultative methylotrophic bacteria and commonly found in association with plants [26]. Ogunade et al [27] found that the abundance of *Methycobacterium* is positively correlated with the pH value in silages, and could be reduced by the microbial inoculants.

*Lactococcus* and *Lactobacillus* became the dominant genera after ensiling, albeit their community abundances on fresh Sudangrass were lower than 0.50%. Upon ensiling, the environment in silos shifted from a stable-state to a new state in which better-adapted microorganisms started to dominate the fermentation. Genus *Lactococcus* was predominant during the early 3 days of fermentation, and subsequently it was replaced by *Lactobacillus* on day 60 of ensiling. This was in accordance with the findings of Keshri et al [24], who reported that while the bacterial diversity varied dramatically at the beginning of fermentation, *Lactobacillus* always dominated the final silage due to their acid-tolerant characteristics.

*Weissella* accounted for a relatively higher abundance on day 3 of ensiling. *Weissella* is strictly heterofermentative, producing a mixture of lactic acid and acetic acid by metabolizing water soluble carbohydrate, and it is the prevailing identifiable genus in untreated crop silage [28]. Furthermore, the relative abundance of *Enterobacter* was increased during the ensiling. Santos et al [29] reported that most of the *Enterobacter* detected in silages are non-pathogenic. Nevertheless, their development is undesirable because they compete with LAB for water soluble carbohydrate at the early stage of fermentation. Silva et al [30] also found that *Enterobacter* are adverse to fermentation quality because they facilitate the production of ammonia nitrogen and decelerate the acidification of fermentation.

The negative relationship between *Lactobacillus* and water soluble carbohydrate contents proved that *Lactobacillus* was mainly responsible for the depletion of water soluble carbohydrate contents during the late stage of ensiling. *Lactobacillus* were positively correlated with volatile fatty acids and negatively related to Enterobacteriaceae populations. This result was similar with the findings of Cao et al [31], who reported that the volatile acids produced by *Lactobacillus* can restrict the growth of harmful microorganisms (e.g. yeasts, molds, pathogenic bacteria).

### Predicted metabolic pathways on three levels

This study firstly revealed the KEGG metabolic pathways of bacterial community in fresh Sudangrass and Sudangrass silages. On the first pathway level, metabolism was the predominant metabolic pathway, which proved that the fermentative process of silage is mainly regulated by bacterial activities via different metabolic pathways to convert fermentable substrates to various metabolites. The metabolic pathways involved in fermentation quality on the second pathway level were the metabolism of amino acid, carbohydrate, energy, nucleotide, vitamins and cofactors [32]. As essential substances in plants, amino acids are crucial to promote the primary metabolism and plant protein synthesis. The primary metabolites are some substances produced by microbes via metabolic activities, such as amino acids. The amino acid metabolism in this study was obviously suppressed after ensiling, which was related to the rapid decrease in pH values during early stage of ensiling. This was probably because the lower pH in Sudangrass silages inhibited the amino acid metabolism resulted from some undesirable bacteria [33]. The carbohydrate metabolism mainly contained gluconeogenesis and glycolysis metabolism [34]. The carbohydrate metabolism herein was gradually enhanced with the ensiling process, whilst the LAB numbers were reduced. It was speculated that the expression of carbohydrate metabolism pathway was more correlated with some undesirable bacteria during the ensiling of Sudangrass.

The malate decarboxylation, amino acid decarboxylation and arginine deamination are three major energy metabolism pathways in LAB, which could promote the production of lactic acid during the ensiling [35]. In the present study, the energy metabolism was inhibited after ensiling. This was contrary to the findings of Xu et al [36], who reported that the energy metabolism was estimated to be promoted in quality silages. Hence, the role of energy metabolism during ensiling needs to be further studied by other omics methods (e.g. proteomics, metabolomics). Notably, the metabolism of cofactors and vitamins was decreased during the ensiling of Sudangrass. Previous studies found that the use of LAB inoculants could directly promote the production of vitamins (e.g. α-tocopherol and β-carotene) in silages [32,37]. It was suggested that the metabolism of cofactors and vitamins in Sudangrass silages could be accelerated by adding some specified LAB inoculants. The nucleotide metabolism was facilitated after ensiling. Kilstrup et al [38] reported that nucleotides can be used to synthesize DNA and provide the main energy for cellular processes. It demonstrated that LAB strains in red clover silage multiplied rapidly at the initial stage of ensiling, which was in accordance with the higher LAB populations during the early 7 days of ensiling.

The carbohydrate metabolism and amino acid metabolism were further investigated on the third pathway level. Most carbohydrate metabolic pathways were accelerated with the ensiling time. This agreed with our findings that the WSC contents were constantly consumed by various microorganisms during the ensiling. In contrast, the metabolism pathways of C5-Branched dibasic acid, inositol phosphate, butanoate, propanoate, glyxylate and dicarboxylate, and citrate cycle (TCA cycle) were not affected or even suppressed after ensiling. The inhibition of citrate cycle (TCA cycle) metabolism may be related to the consumption of oxygen, because the TCA cycle must be carried out under aerobic conditions. Without oxygen, the removed hydrogen ions cannot enter the respiratory chain for complete oxidation [39]. Most amino acid metabolic pathways were markedly inhibited after ensiling, which was consistent with the relatively lower NH_3_-N contents in Sudangrass silages. This was probably because the rapid decline in pH values during the early stage of fermentation restricted the protein degradation by undesirable microorganisms [21].

### Some key enzymes in various metabolic pathways

Lactic acid bacteria can be classified into two groups on the basis of their fermentation end products: homofermentative and heterofermentative. Homofermentative LAB virtually produce only lactic acid, whereas other products are produced by heterofermentative LAB accompanied with lactic acid. In the metabolism of homofermentative LAB, glucose is metabolized to lactic acid via the Embden-Meyerhof pathway (EMP). On the other hand, heterofermentative LAB possess the pentose phosphate pathway (PPP) [40]. It is well known that the hexokinase, 1-phosphofructokinase and pyruvate kinase are the most important enzymes in the EMP pathway. The promotion of 1-phosphofructokinase and pyruvate kinase and the inhibition of hexokinase after ensiling indicated that the lactic acid fermentation occurred in Sudangrass silages was more related to the 1-phosphofructokinase and pyruvate kinase rather than hexokinase. The glucose-6-phosphate dehydrogenase was mainly involved in the PPP pathway. Thus, the significant decrease of glucose-6-phosphate dehydrogenase proved that the heterofermentative process was restricted during the ensiling of Sudangrass, which was opposite to the increased trend of pentose phosphate pathway on the third pathway level. This was probably because the complicated PPP pathway was involved in various enzymes, and the glucose-6-phosphate dehydrogenase could not typically represent this pathway. The enhancement of D-lactate dehydrogenase and L-lactate dehydrogenase was consistent with the massive accumulation of lactic acid during the entire ensiling process. Notably, lactic acid is formed as the end product of glycolysis in LAB. It means that LAB during growth face an environment that continually increases in acidity. It is therefore reasonable to assume that low pH condition could induce other systems in LAB to buffer lactic acid acidity, such as arginine deimination (ADI pathways) [41]. Thus, the up-regulation of arginine deiminase after ensiling may result from the increase of acidity in the silage environment.

## CONCLUSION

The Sudangrass silages showed good fermentation quality, as indicated by higher lactic acid contents and ratios of lactic acid to acetic acid, and lower pH values and contents of butyric acid and acetic acid. The dominant genus *Lactococcus* on day 3 was replaced by *Lactobacillus* on day 60. The metabolism of amino acid, energy, cofactors and vitamins was reduced, and metabolism of nucleotide and carbohydrate was promoted after ensiling. Most amino acid metabolic pathways were inhibited after ensiling, while most carbohydrate metabolic pathways were promoted with the ensiling process. The 1-phosphofructokinase and pyruvate kinase of bacterial community played important roles in accelerating the lactic acid fermentation. Overall, the high-throughput sequencing technology combined with 16S rRNA gene-predicted functional analyses revealed the differences during the ensiling of Sudangrass not only for distinct bacterial community but also for specific functional metabolites. This result could provide a comprehensive insight into bacterial community and functional profiles to further improve the silage quality.

## Figures and Tables

**Table 1:** Chemical and microbial compositions of Sudangrass prior to ensiling

Items	Sudangrass
Chemical compositions	
pH	6.09±0.02
Dry matter (g/kg FW)	204±1.41
Water soluble carbohydrates (g/kg DM)	87.6±0.82
Buffering capacity (mEq/kg DM)	218±1.79
Neutral detergent fiber (g/kg DM)	648±4.01
Acid detergent fiber (g/kg DM)	384±2.18
Acid detergent lignin (g/kg DM)	59.3±1.13
Crude protein (g/kg DM)	32.7±1.15
Microbial compositions	
Lactic acid bacteria (log_10_ cfu/g FW)	4.78±0.10
Aerobic bacteria (log_10_ cfu/g FW)	7.47±0.03
Yeasts (log_10_ cfu/g FW)	4.34±0.02
Enterobacteriaceae (log_10_ cfu/g FW)	7.27±0.09

Data are mean values and standard deviations for triplicate samples.

DM, dry matter; FW, fresh weight; mEq, milligram equivalent; cfu, colony-forming units.

**Table 2:** Changes in fermentation characteristics and microbial compositions during the ensiling of Sudangrass

Items	Ensiling days (d)						SEM

	1	3	7	15	30	60	
Chemical compositions (g/kg DM)							
pH	5.88^a^	4.85^b^	4.66^c^	4.25^d^	3.91^f^	4.05^e^	0.162
Lactic acid	5.78^f^	19.5^e^	25.4^d^	37.4^c^	59.1^b^	62.6^a^	2.99
Acetic acid	1.86^e^	5.16^d^	6.74^b^	7.50^a^	6.28^c^	5.20^d^	0.441
LA/AA	3.10^d^	3.78^d^	3.77^d^	4.98^c^	9.42^b^	12.0^a^	0.81
Propionic acid	0.580	0.680	0.467	0.557	0.713	0.743	0.0314
Isobutyric acid	1.06^d^	1.14^cd^	1.25^c^	1.56^b^	1.76^a^	1.67^ab^	0.073
Butyric acid	0.79^c^	0.92^c^	1.58^b^	1.68^b^	1.83^a^	1.71^ab^	0.102
Ethanol	17.5^e^	30.9^d^	34.4^c^	38.1^b^	42.5^a^	40.0^b^	2.01
VFAs	4.29^f^	7.91^e^	10.0^c^	11.3^a^	10.6^b^	9.32^d^	0.561
Dry matter (g/kg FW)	195^a^	185^b^	182^b^	174^c^	168^cd^	166^d^	2.5
NH_3_-N (g/kg TN)	12.5^f^	25.4^e^	32.6^d^	38.2^c^	45.3^b^	56.7^a^	1.44
WSC	67.5^a^	39.0^b^	31.4^c^	24.0^d^	14.2^e^	6.79^f^	2.763
Microbial compositions (log_10_ cfu/g FW)							
Lactic acid bacteria	7.45^c^	9.41^a^	9.71^a^	8.44^b^	7.35^c^	6.22^d^	0.304
Enterobacteriaceae	7.49^a^	7.29^a^	6.28^b^	5.36^c^	4.65^d^	4.07^e^	0.312
Yeasts	4.72^a^	4.15^cd^	4.01^cd^	3.91^d^	4.22^bc^	4.49^ab^	0.073

SEM, standard error of the mean; DM, dry matter; VFAs, volatile fatty acids; FW, fresh weight; LA/AA, ratio of lactic acid to acetic acid; NH_3_-N, ammonia nitrogen; TN, total nitrogen; WSC, water soluble carbohydrate; cfu, colony-forming units.

a–f Means within the same row with different letters differ significantly from each other (p<0.05).

**Table 3:** Richness and diversity indexes of bacterial communities in fresh Sudangrass and Sudangrass silages during ensiling

Items	SDFM	SD-3	SD-60
OTUs	76.40	79.40	66.47
Shannon	2.885	2.068	1.872
Chao1	75.00	79.33	61.03
Sobs	73.00	71.00	58.67
Simpson	0.0942	0.1926	0.2096
Ace	74.13	80.64	63.15
Coverage	0.9999	0.9997	0.9998

SDFM, fresh Sudangrass; SD-3, Sudangrass silage after 3 days of ensiling; SD-60, Sudangrass silage after 60 days of ensiling; OTUs, operational taxonomic units.
